# FAMILY DEMENTIA CAREGIVERS’ ASSISTANCE WITH ORAL HYGIENE CARE IN CHINESE AMERICAN COMMUNITIES IN NYC

**DOI:** 10.1093/geroni/igae098.1428

**Published:** 2024-12-31

**Authors:** Weiyu Mao, Bei Wu, Yaolin Pei

**Affiliations:** University of Nevada, Reno, Reno, Nevada, United States; New York University, New York, New York, United States; University of Texas at Austin, Texas, United States

## Abstract

Oral hygiene care is instrumental in maintaining good oral health. As dementia progresses, individuals face challenges performing adequate oral hygiene care and become dependent on their caregivers. The role of family caregivers in assisting with oral hygiene care for persons with dementia in the community becomes increasingly critical. This study explored the association between caregiving characteristics, care recipients’ oral health needs, and assistance in oral hygiene care among caregivers in Chinese American communities. Data came from a pilot study on Chinese dementia caregivers in New York City collected between November 2021 and June 2022. Purposive sampling was used to recruit family caregivers to participate in a survey (online or via telephone). Current caregivers (n = 76) were included in the present study. Caregiver assistance with oral hygiene care was measured by assistance with toothbrushing (yes or no) and assistance with flossing (yes or no). Results from group comparisons suggest that female caregivers tended to assist with toothbrushing, and caregivers with an average of 2.4 years in providing care tended to assist with flossing. Logistic regressions show that care recipients with tooth pain were 5.6 times more likely to receive assistance with toothbrushing. Moreover, care recipients with more natural teeth were 1.1 times more likely to receive assistance with toothbrushing. Care recipients with severe dementia were 98% less likely to receive assistance with flossing. It is important to raise awareness and address challenges among dementia caregivers regarding the importance of maintaining adequate oral hygiene care and, subsequently, promoting optimal oral health.

